# Mortality and predictors of acute kidney injury in adults: a hospital-based prospective observational study

**DOI:** 10.1038/s41598-021-94946-3

**Published:** 2021-08-02

**Authors:** Abinet Abebe, Kabaye Kumela, Maekel Belay, Bezie Kebede, Yohannes Wobie

**Affiliations:** 1 School of Pharmacy, College of Medicine and Health Science, Mizan-Tepi University, Mizan, Ethiopia; 2 School of Pharmacy, Institute of Health, Jimma University, Jimma, Ethiopia; 3 Jimma Medical Center, Jimma, Ethiopia

**Keywords:** Nephrology, Urology

## Abstract

Acute kidney injury (AKI) is a major global public health problem. It is expensive to manage and associated with a high rate of prolonged hospitalization and in-hospital mortality. Little is known about the burden of acute kidney injury in moderate to low-income countries. We aim to assess predictors of in-hospital mortality among AKI patients admitted to the medical ward. We prospectively identified patients meeting kidney disease improving global outcomes (KIDGO) AKI definitions from April to August 2019. Patients with underlying CKD and patients hospitalized for less than 48 h were excluded. The Cox regression model was fitted to identify predictors of mortality and statistical significance was considered at the *p*-value of less than 0.05. A total of 203 patients were enrolled over 5 months. Out of this, 121(59.6%) were males, 58(28.6%) were aged greater than 60 years, and 141(69.5%) had community-acquired acute kidney injury. The most common causes of AKI were Hypovolemia 99(48.77%), Glomerulonephritis 51(25.11%), and sepsis 32(15.79%). The overall in-hospital mortality rate was 12.8%. Stage 3 AKI (AHR = 9.61, 95% CI 1.17–28.52, *p* = 0.035), duration of AKI (AHR = 7.04, 95% CI 1.37–36.08, *p* = 0.019), length of hospital stay (AHR = 0.19, 95% CI 0.05–0.73, *p* = 0.012), and hyperkalemia (AHR = 3.61, 95% CI 1.12–11.71, *p* = 0.032) were significantly associated with in-hospital mortality. There is a high rate of acute kidney injury-related in-hospital mortality in adult patients admitted to the medical ward. The severity of AKI, hyperkalemia duration of AKI, and a short length of hospital stay were predictors of 30-days in-hospital mortality. Most of the causes of AKI are preventable and patients may benefit from early identification and treatment of these reversible causes.

## Introduction

Acute kidney injury (AKI) is a complex clinical syndrome that arises in response to many etiologies. It is a major global public health problem that can occur both in the community and hospital settings^1^. It is expensive to manage and associated with high-rate prolonged hospitalization and in-hospital mortality. The in-hospital mortality rate is reported at 24% and increases with the severity of the disease^2,3^. Based on United Kingdom National Institute for Health and Care Excellence (UK-NICE) published a report on AKI, early identification, and treatment with attention to hydration and medications could avoid up to 42,000 deaths from AKI each year^4^.

Despite advances in AKI definition and opportunities of prevention, renal replacement therapy, and supportive measures, in-hospital mortality remains high. There is still a lack of awareness of the disease and its long-term impacts. AKI affects over 13 million people and results in 1.7 million deaths each year around the world. Even a mild form of AKI is associated with a 50% higher risk of death. It also imposes a significant burden on society in terms of chronic kidney disease and end-stage kidney failure^5–7^.

The burden of acute kidney injury is high especially, in developing countries with limited resources for the care of these patients once the disease progresses to end-stage kidney failure. Most of the data on acute kidney injury are derived from high-income countries, in low to moderate-income countries; the impact of AKI is almost completely unknown^1,8,9^.

Failure of early recognition of AKI with active monitoring of renal function leads to the development of acute renal injury or end-stage renal disease (ESRD). Patients with confirmed AKI are treated inappropriately and the treatment itself is costly in resource-limited settings especially; if progress to ESRD. Due to limited nephrology services, the high morbidity and mortality associated with acute kidney injury in Ethiopia are challenging to the community. Government-run dialysis services in Ethiopia and access to dialysis are limited and some patients are referred to private dialysis facilities where treatment is limited by the inability to cover the costs of treatment^10,11^.

Limited data is available regarding renal function tests in hospitalized patients, risk factor recognition, outcomes, and associated factors of AKI in Ethiopia^10,12^. Therefore, this study was aimed to assess predictors of in-hospital mortality among AKI patients admitted to the medical ward of Jimma Medical Center, Southwest Ethiopia.

## Methods and materials

### Study design and population

A hospital-based prospective observational study was conducted from April to August 2019 at the medical ward of Jimma Medical Center, Southwest Ethiopia. The internal medicine department of the hospital has over 80 beds for inpatients and has two renal units^13^. All adult patients with the diagnosis of acute kidney injury were consecutively enrolled in the study and followed till discharge from the hospital or death. Single population proportion formula was used to determine the sample size. Patients with age ≥ 18 years, patients with confirmed AKI, and patients who were willing to sign written informed consent were included in the study. On the other hand; patients who cannot be able to give the required information, hospitalized for less than 48 h, and those patients with underlying chronic kidney disease (CKD) were excluded from the study.

### Sample size determination and sampling technique

The proportion of patients with AKI-related mortality was 31.2%, as reported from an earlier study done in Sudan among adult acute kidney injury patients^14^. The required sample size was determined by using single population proportion formula:

$${\text{n}} = \frac{{({\text{Z}}\upalpha {/}2)^{2} {\text{p}}(1 - {\text{p}})}}{{{\text{d}}^{2} }}$$

where n = the desirable sample size, Za/2 = 1.96 (the critical value at 95% level of significance), p = 0.312 (proportion of patients with AKI related mortality), d = 0.05 (acceptable marginal error) and 1 − p = proportion of patients that do not possess the character of interest. Replacing with values results in:

$${\text{n}} = \frac{(1.96)2\;(0.312)(0.668)}{{(0.05)\;2}} = 330$$

The total number of adults patients with AKI admitted to the medical ward of the hospital during the last 6 months were 212. The number of the source population (N) was 212, which is below 10,000. Correction formula issued to determine final minimum sample size (nf).

$${\text{nf}} = \frac{{\text{n}}}{{1 + {\text{n/N}}}} = \frac{330}{{1 + 330{/}212}} = 129$$

By adding 10% drop out, the final minimum sample size was 142.

The consecutive sampling technique was used to collect data from patients who met the inclusion criteria.

### Data collection and procedures

Data abstraction checklist and structured questionnaire were developed from a review of the literature^14–16^. We collected information about patient demographics, diagnosis, comorbidities (hypertension, diabetes, heart failure, chronic liver disease), laboratory and other investigation results (repeated measures of creatinine, urea, electrolytes, complete blood count, and liver function tests). The data collection tool was first pretested on 10 (5%) patients at Mizan-Tepi University teaching hospital to validate and check the consistency, applicability, and understandability of the questionnaire and data abstraction format before the actual data collection.

### AKI and outcomes

AKI was defined according to the KDIGO 2012 AKI criteria (1) an increase in Scr by ≥ 0.3 mg/dl within 48 h, (2) an increase in Scr to ≥ 1.5 times baseline occurred within the prior 7 days, (3) urine volume < 0.5 ml/kg/h for 6 h. AKI was staged based on KIDGO criteria as follows. Stage 1 AKI: Scr increase > 0.3 mg/dl or increase to 1.5–1.9 times from baseline OR urine output < 0.5 ml/kg/h for 6–12 h. Stage 2 AKI: Scr increase 2.0–2.9 times from baseline OR urine output < 0.5 ml/kg/h for ≥ 12 h. Stage 3 AKI: Scr increase > 3.0 times from baseline OR increase in Scr to > 4.0 mg/dl, OR urine output < 0.3 ml/kg/h for ≥ 24 h OR Anuria for ≥ 12 h, OR initiation of RRT^1^. The first documented serum creatinine on admission was used as a baseline for hospital-acquired AKI^1^. For community-acquired AKI, (1) Prehospital record of creatinine within 7 days–3 months of hospital admission, whichever available, or (2) The minimum and or most recent value of admission Serum creatinine was used as baseline^17–19^. Comorbidities were defined as any chronic medical conditions that coexist with AKI. Renal recovery was defined as a return to baseline kidney function at hospital discharge. Duration of AKI was defined as the number of days from the first day the patient met the AKI criteria until they no longer did. Prolonged length of hospital stay was defined as hospital stays greater than 7 days^20,21^.

*Outcomes* The main outcome variable was all-cause mortality. Mortality was defined as death before hospital discharge. Other outcomes included recovery of kidney function at discharge (improved), non-recovery AKI, referral, and self-discharge. Patients were followed starting from diagnosis of AKI to occurrence of the event.

### Statistical analysis

Epi data version 4.4.2 was used to enter, encode and clear data, and SPSS version 21 was used for analysis. Descriptive statistics, such as frequency, percentage, mean, median, and standard deviation were used to summarize patients’ baseline clinical characteristics. Frequency and proportion were used for categorical data, while the mean and standard deviation for continuous variables. Crosstabs were used for the comparison of proportions of categorical variables. The Cox regression model was fitted to determine independent predictors of mortality. Statistical significance was considered at the *p*-value of less than 0.05 on multivariate analysis.

### Ethical approval and consent to participate

The institutional review board of Jimma University, institute of health, research ethics unit approved the study (Reference Number IHRPGA-574-2019), and a letter of permission was forwarded to the administration of Jimma Medical Center. Written informed consent was secured from all participants and the collected data was kept confidential. All methods were carried out in accordance with the relevant guidelines and regulations.

### Consent for publication

Not applicable.

## Results

### Baseline characteristics of patients

A total of 234 AKI patients were identified but only 203 patients were recruited consecutively over 5 months with a response rate of 97.5%. These 31 patients were excluded because 18 patients had underlying CKD, 8 patients were hospitalized for less than 48 h, and 5 patients were not willing to participate in the study. Out of 203 patients, 141(69.5%) had community-acquired AKI (CA-AKI). Males accounted for 121(59.6%) of all AKI admissions. The mean age was 48.98 ± 14.97 years (range 18–82 years). Patients aged 60 years and above were 58(28.6%). Baseline characteristics of patients are summarized in Table 1.

**Table 1:** Baseline clinical characteristics of AKI patients at JMC (n = 203).

Variables	Category	Frequency	Percent (%)
Sex	Male	121	59.6
	Female	82	40.4
Age	18–30	28	13.8
	30–60	117	57.6
	≥ 60	58	28.6
Type of AKI	CA-AKI	141	69.5
	HA-AKI	62	30.5
Hypertension	Yes	131	64.5
	No	72	35.5
Heart failure	Yes	59	29
	No	144	71
Anemia	Yes	20	10
	No	183	90
Diabetes	Yes	14	6.9
	No	189	93.1
HIV	Yes	10	5
	No	193	95
Chronic liver disease	Yes	4	2
	No	199	98

AKI, Acute kidney injury; HIV, Human immune virus; HAAKI, Hospital-acquired AKI; CAAKI, Community-acquired AKI; JMC, Jimma Medical Center.

### Laboratory values

The mean levels of creatinine at diagnosis and discharge were 5.31 ± 3.22 mg/dl and 4.56 ± 2.84 mg/dl respectively. Serum electrolyte assessment was done for 133(65.5%) patients. The mean levels of potassium at diagnosis of AKI and discharge were 5.19 ± 1.83 mEq/L and 4.51 ± 1.13 mEq/L respectively. On the other hand, CBC was done for 24% of patients, 14% of patients had anemia and 8% had leukocytosis. The result for selected laboratory values is shown in Table 2.

**Table 2:** Laboratory values for AKI patients at JMC (n = 203).

Laboratory value	Time	Mean ± SD
BUN	At diagnosis	107.97 ± 91.94 mg/dl
	At discharge	97.49 ± 81.25 mg/dl
Serum creatinine	At diagnosis	5.31 ± 3.22 mg/dl
	At discharge	4.56 ± 2.84 mg/dl
Sodium	At diagnosis	132.02 ± 6.15 mEq/L
	At discharge	135.14 ± 3.71 mEq/L
Potassium	At diagnosis	5.19 ± 1.83 mEq/L
	At discharge	4.51 ± 1.13 mEq/L
Ionized calcium	At diagnosis	1.02 ± 0.19 mEq/L
	At discharge	1.26 ± 0.13 mEq/L
Chloride	At diagnosis	109.96 ± 10.31 mEq/L
	At discharge	104.40 ± 11.74 mEq/L
Hemoglobin	At diagnosis	9.25 ± 2.46 mg/dl
	At discharge	11.52 ± 3.14 mg/dl
WBC	At diagnosis	11.22 ± 6.61 × 10 * 3
	At discharge	10.33 ± 3.58 × 10 * 3
RBS	At diagnosis	153.71 ± 35.47 mg/dl
	At discharge	138.33 ± 26.12 mg/dl

BUN, blood urea nitrogen; WBC, white blood cell; RBS, random blood sugar.

Based on KIDGO criteria, eighty-six patients (42.4%) had stage 3 AKI. Most patients (87.2%) had prolonged lengths of hospital stay. The median length of hospital stay was 11 days. More than half (57.2%) of patients had persistent AKI (AKI duration ≥ 7 days). The mean AKI duration was 6.59 days. From patients with urine output assessment at diagnosis of AKI, 29.4% had oliguric and 70.6% had non-oliguric AKI. The duration of oliguria was 6 ± 2 days. At discharge, only 64.5% had urine output assessment. From this, 3.53% were anuric and 7.07% were oliguric. Clinical characteristics of AKI patients are outlined in Table 3.

**Table 3:** Clinical characteristics of AKI patients at JMC (n = 203).

Variable	Category	Frequency	Percent (%)	No of death (%)
Stage of AKI	Stage 1	69	34	4(1.97)
	Stage 2	48	23.6	7(3.4)
	Stage 3	86	42.4	15(7.4)
Length of hospital stay	≤ 7 days	26	12.8	14(6.89)
	> 7 days	177	87.2	12(5.91)
Duration of AKI	1–2 days	35	17.2	9(4.43)
	3–6 days	52	25.6	5(2.46)
	≥ 7 days	116	57.2	12(5.91)
Need for RRT	Yes	20	10	11(5.4)
	No	183	90	15(7.4)
Urine output	Oliguric	43	29.4	7(3.44)
	Nonoliguric	103	70.6	4(1.9)

JMC, Jimma Medical Center; AKI, acute kidney injury; RRT, renal replacement therapy.

### Causes of AKI

The cause of AKI and death from each cause of AKI are outlined in Table 4. More than half of patients (52%) had pre-renal AKI. The other 14% and 4.5% had intrinsic and post-renal AKI respectively. The remaining 29.5% had unspecified AKI. The most common causes of AKI were hypovolemia (mainly GI loss) 49%, glomerulonephritis including nephritic syndrome 25.11%, and sepsis 15.79%. Four patients (1.97%) had cervical cancer (obstructive nephropathy) as the cause of AKI.

**Table 4:** Causes of AKI and death from each cause, JMC (n = 203).

Cause of AKI	Frequency	Percent (%)	Death from each cause of AKI (%)
Hypovolemia	99	48.77	8(3.94%)
Glomerulonephritis including NS	51	25.11	9(4.42%)
Sepsis	32	15.79	4(1.97%)
Nephrotic syndrome	10	4.92	1(0.5%)
BPH	5	2.46	0
Cervical cancer (obstructive nephropathy)	4	1.97	4(1.97%)
Hydronephrosis	2	0.98	0

AKI, acute kidney injury; BPH, benign prostatic hyperplasia; NS, nephritic syndrome.

### Outcomes of AKI

#### Mortality

The overall in-hospital rate of mortality was 12.8% (n = 26). Glomerulonephritis and hypovolemia were the most common causes of AKI which accounted for more than 65% of deaths (Table 4). Of the 26 patients, 15(57.7%) had stage 3 AKI and, 14(53.8%) had a short length of hospital stay. On multivariate Cox regression; patients with AKI duration of ≥ 7(AHR = 7.046; CI 1.37–36.08, *p* = 0.019), stage 3 AKI (AHR = 9.60; CI 1.175–28.52, *p* = 0.035) and hyperkalemia (AHR = 3.61, CI 1.17–11.71, *p* = 0.032) were factors associated with 30-days in-hospital mortality. Patients with a length of hospital stay greater than or equal to 7 days (AHR = 0.19, CI 0.05–0.73, *p* = 0.012) had an 81% lower risk of death when compared to patients hospitalized for less than 7 days. The results for bivariate and multivariate cox regression analysis are outlined in Table 5.

**Table 5:** Bivariate and multivariate cox regression analysis of predictors of mortality (n = 26).

Variables	Category	Outcome		HR (95% CI)	*P*-value	AHR (95% CI)	*P*-value
		Survive	Death				
		177	26				
Hyperkalemia	No	155	10	Reference		Reference	
	Yes	22	16	12.1 (5.23, 27.99)	0.000	3.61(1.12, 11.71)	0.032*
Stage of AKI	Stage 1	65	4	Reference		Reference	
	Stage 2	41	7	1.30(0.08, 20.88)	0.851	1.7(0.10, 29.04)	0.691
	Stage 3	71	15	12.27(2.74, 49.8)	0.003	9.6(1.17, 28.52)	0.035*
Sepsis	No	153	18	Reference		Reference	
	Yes	24	8	8.1(3.69–17.79)	0.000	3.12(0.95, 10.17)	0.059
Anemia	No	157	18	Reference		Reference	
	Yes	20	8	9.47(3.14, 28.89)	0.000	0.40(0.07, 2.109)	0.281
LOHS (days)	≤ 7	9	17	Reference		Reference	
	> 7	168	9	0.05(0.02, 0.13)	0.000	0.19(0.05, 0.73)	0.012*
Dur–AKI(days)	1–2	33	2	Reference		Reference	
	3–6	50	2	0.32(0.04, 2.3)	0.251	1.09(0.12, 10.05)	0.935
	≥ 7	94	22	4.28(1.05, 18.3)	0.049	7.046(1.37, 36.08)	0.019*
Need for RRT	No	167	16	Reference		Reference	
	Yes	14	6	12.4(5.04, 30.78)	0.000	2.15(1.04, 4.76)	0.061
Age in years	18–30	24	4	Reference		Reference	
	30–60	108	9	2.35(0.29, 18.62)	0.416	1.5(0.17, 12.01	0.725
	≥ 60	45	13	7.93(1.05, 59.95)	0.045	3.03(0.35, 26.45)	0.316

AKI, acute kidney injury; HR, hazard ratio; AHR, adjusted hazard ratio; CI, confidence interval; LOHS, length of hospital stay; Dur-AKI, duration of AKI; RRT, renal replacement therapy.

*Statistically significant.

*Other outcomes* One hundred eight (53.2%) patients were discharged improved (Return to baseline kidney function). Of those patients discharged with improved renal function, 13% had sustained reversal and the other 40.2% had a late reversal. The average duration of late reversal was 11.30 days. The minimum and maximum duration of late reversal were 8 and 21 days respectively. Patients discharged with non-recovery AKI were 62 (30.5%). The remaining 4(2%) patients were referred for further investigation and treatment and 3(1.5%) patients were self-discharged.

**Figure 1:**
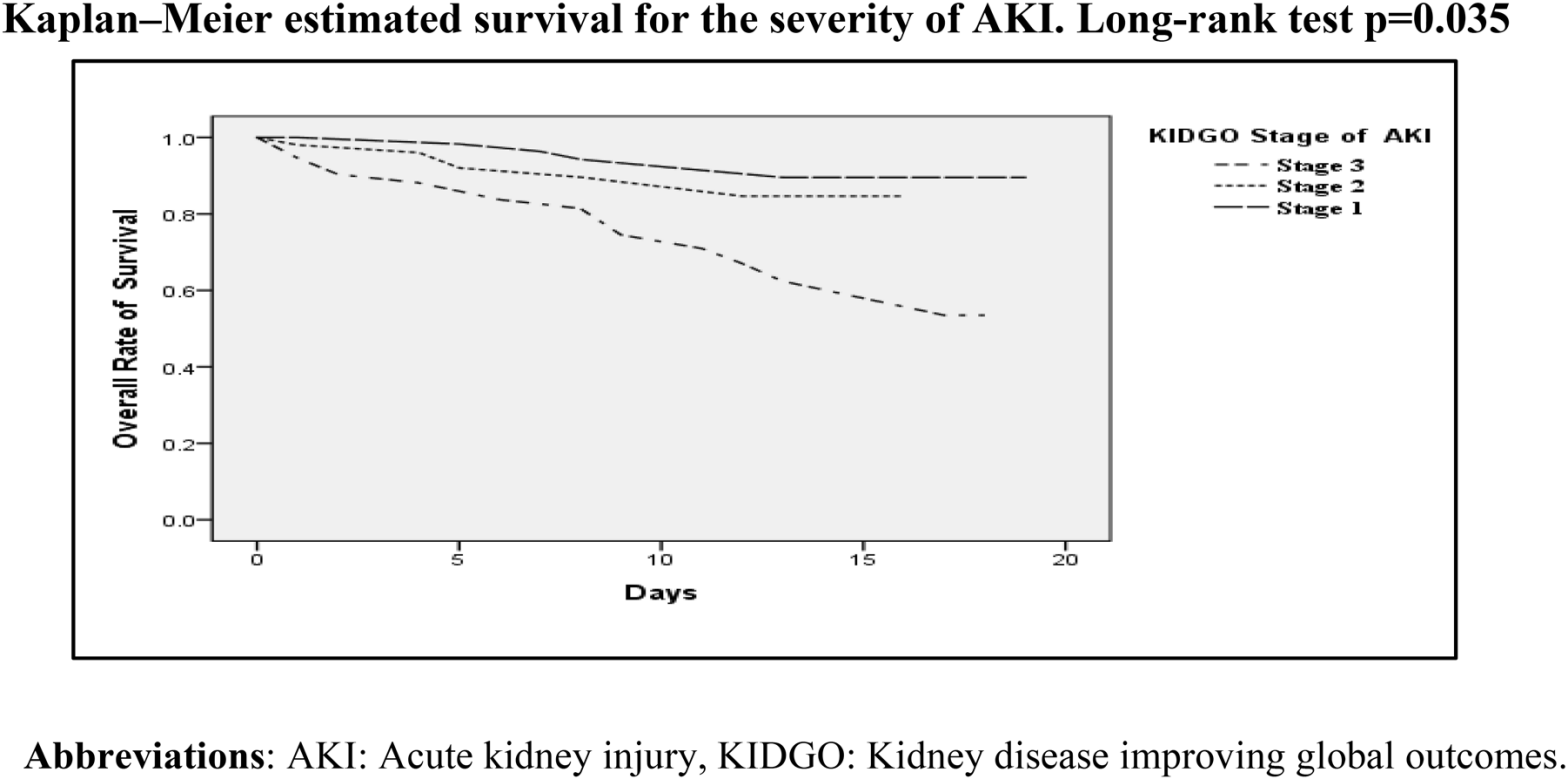
Kaplan–Meier analysis demonstrating survival probability in patients with stage 1, stage 2, and stage 3 AKI up to 20 days.

The probability of survival among the proportion of patients with stage 3 AKI decreases with time compared to patients with stage 1and stage 2 AKI (Fig. 1).

## Discussion

The pattern of acute kidney injury (AKI) in this study is similar to that reported in developing countries. In the present study; community-acquired AKI accounted for 69.5% of cases, almost within a range of results reported from a study done in Sudan^14^. AKI in developing countries is believed to be community-acquired, occur between 70 and 90% of cases^9,22^. Lack of resources for early detection and treatment, patient’s late arrival to health facilities with advanced-stage disease, and delay in diagnosis can contribute to the increase in the burden of AKI in developing countries.

The overall in‑hospital mortality of AKI in our study (12.8%) is much lower compared to findings from other studies, 22.6% in Egypt, 36.9% in Cameroon, 44.4% in Malawi and, 20.3% in Singapore^6,16,23,24^. The variation with these studies might be due to; the difference in patient’s site of admission (i.e. ICU vs general medical ward), study design, and underlying disease of patients. Our study excludes patients with underlying CKD, which is a known predictor of in-hospital mortality in patients with AKI. The result of this study was comparable with other studies, 13% in Egypt^25^ and 10.8% in Sanford-USA^26^.

Among those who died, the severity of AKI as per KDIGO staging, presence of hyperkalemia, duration of AKI, and length of hospital stay were significant predictors of in-hospital mortality. Patients with stage 3 AKI had a higher risk of 30-days in-hospital mortality when compared with patients with a less severe stage of the disease (Table 5). The reason might be due to the patient’s' delayed admission to the hospital with an advanced stage of the disease and missed attempts to prevent the progression of the disease. This is consistent with several other studies done in Egypt, Brazil, and India respectively^25,27,28^. Another study also showed that even patients with a 25–50% increase in peak serum creatinine levels from baseline have an increased risk of mortality. Stage of AKI is reported to be a predictor for mortality and decreased kidney function, correlate with short term and even longer-term adverse outcomes^1,29^.

In this study; patients with a short length of hospital stay (≤ 7 days) were shown to have a high risk of in-hospital mortality. This is similar to a result reported from a study done in Nigeria^30^. This may be due to limited resources to detect AKI early in health centers and primary hospitals, which leads to late referral and admission of patients with a severe stage of AKI, usually with complications, and died early on hospital admission.

Persistent AKI can be associated with worse clinical outcomes include increased length of stay, time on the ventilator, and days with cardiovascular failure^31^. We found that patients with persistent AKI had an increased risk of in-hospital mortality. This is similar to findings from other studies where persistent AKI is shown to be associated with increased risk of mortality^25,32^. Studies suggest AKI duration along with the stage of AKI should be used as a parameter for the prediction of worse outcomes especially. This may help physicians in preventing in-hospital and post-discharge mortality especially in critically ill, high risk patients^33^.

The in-hospital mortality rate of patients with hyperkalemia was shown to be 30.7%^34^. In this study, hyperkalemia patients had higher mortality rates than patients with normokalemia. Other studies also showed that, compared to patients with normokalemia, hyperkalemia was associate with a higher percentage of death^28,35^. Therefore, early identification of high-risk patients and maintaining serum potassium level within a safe range may help to avoid the risk of hyperkalemia related-mortality.

Our study has some limitations. First, urine output assessment was done in around 70% of patients. Second, patients with underlying CKD were excluded from the study. Furthermore, no outcome monitoring was performed beyond the period of hospitalization. Therefore, important information, including the incidence of CKD after AKI, mortality rate, and ESRD, and other non-renal outcomes are unknown.

In conclusion**,** the pattern of AKI is consistent with other studies from the developing world. There is a high rate of acute kidney injury-related in-hospital mortality in adult patients admitted to the medical ward. The severity of AKI, hyperkalemia, duration of AKI, and short length of hospital stay were predictors of mortality. Most of the causes of AKI are preventable and patients may benefit from early identification and treatment of these reversible causes.

## Data Availability

The datasets generated and/or analyzed during the current study are available from the corresponding author on reasonable request.
